# Ultraweak Photon Emission from the Seed Coat in Response to Temperature and Humidity—A Potential Mechanism for Environmental Signal Transduction in the Soil Seed Bank

**DOI:** 10.1111/php.12616

**Published:** 2016-08-19

**Authors:** Steven Footitt, Simonetta Palleschi, Eugenio Fazio, Raffaele Palomba, William E. Finch‐Savage, Leopoldo Silvestroni

**Affiliations:** ^1^ School of Life SciencesUniversity of WarwickWarwickshireUK; ^2^ Department of Haematology, Oncology and Molecular MedicineIstituto Superiore di SanitàRomeItaly; ^3^ Department of Fundamental and Applied Sciences for EngineeringSapienza University of RomeRomeItaly; ^4^ The National Institute for Insurance against Accidents at Work (INAIL)RomeItaly; ^5^ Department of Experimental MedicineSapienza University of RomeRomeItaly

## Abstract

Seeds beneath the soil sense the changing environment to time germination and seedling emergence with the optimum time of year for survival. Environmental signals first impact with the seed at the seed coat. To investigate whether seed coats have a role in environmental sensing we investigated their ultraweak photon emission (UPE) under the variable temperature, relative humidity and oxygen conditions they could experience in the soil seed bank. Using a custom‐built luminometer we measured UPE intensity and spectra (300–700 nm) from *Phaseolus vulgaris* seeds, seed coats and cotyledons. UPE was greatest from the internal surface of the seed coat. Seed coat UPE increased concomitantly with both increasing temperature and decreasing relative humidity. Emission was oxygen dependent and it was abolished by treatment with dinitrophenylhydrazine, demonstrating the key role of seed coat carbonyls in the phenomenon. We hypothesize that beneath the soil surface the attenuation of light (virtual darkness: low background noise) enables seeds to exploit UPE for transducing key environmental variables in the soil (temperature, humidity and oxygen) to inform them of seasonal and local temperature patterns. Overall, seed coats were found to have potential as effective transducers of key fluctuating environmental variables in the soil.

## Introduction

Ultraweak photon emissions (UPEs) (also termed low‐level chemiluminescence or biophotons) discovered by Gurwitsch in the 1920s are photons emitted during de‐excitation of high‐energy excited species back to the ground state as a direct result of a chemical reaction 1, 2, 3. There are two types of UPE, spontaneous and induced. Spontaneous UPEs are continuously emitted by almost all metabolically active microbial, plant and animal cells without any external stimuli. They are generally considered to originate from the electronically excited species formed during oxidative metabolic reactions. In contrast, induced UPE is initiated by various biotic (*e.g*. infection) and abiotic (*e.g*. temperature, oxygen and humidity) stresses that lead to production of reactive oxygen species (ROS) and oxidative damage, resulting in ultraweak photon intensities higher than for spontaneous emissions 1.

Ultraweak photon emission covers a broad spectrum of wavelengths (350–1300 nm), with emission intensity ranging from 10^−1^ to 10^4^ photons s^−1^ cm^−2^. The wavelength indicates the electronically excited species responsible for their emission. UPE in the near UVA‐green region (350–550 nm) is associated with the relaxation of triplet excited carbonyls (^3^(C=O)). Triplet excited carbonyls are formed by the oxidative action of ROS on biomolecules, resulting in the decomposition of the high‐energy intermediates dioxetane and tetroxide 1. When the excited carbonyl species are in close proximity to other molecules such as pigments or molecular oxygen, a nonradiative energy transfer can occur with the formation of singlet excited pigments and singlet oxygen (^1^O_2_) (dimol form), which emit in the green‐red region (550–750 nm) and red region (634 and 703 nm), respectively 1, 2. Singlet oxygen is also produced by interaction between hydroxyl (˙OH) and superoxide (O_2_˙^−^) radicals and between excited pigments and molecular oxygen 4. These ROS, triplet excited carbonyls and singlet oxygen, are produced in the oxidation of complex polysaccharides such as cellulose and during lipid peroxidation 5, 6. That ROS are a source of UPE is seen by a reduction in UPE when excited triplet carbonyls are quenched by sorbate anions 7 and when singlet oxygen is quenched by sodium azide 8.

Ultraweak photon emission is implicated in cell‐to‐cell signaling; for example, neutrophils stimulated to undergo respiratory burst showed increased UPE and induced activation of a neighboring population chemically separated, but optically coupled 9, 10. A number of other studies have shown impacts on cell growth and orientation where chemical communication was prevented and only optical coupling *via* glass or quartz barriers were possible. The effect was lost when these barriers were blinded by metal films 10, 11. However, there is still debate about how UPEs are perceived by nonspecialized cells especially in the presence of a low signal‐to‐noise ratio resulting from noise in the form of ambient light that can be several magnitudes higher 9.

In rice and wheat seeds, UPE showed an initial spike on the addition of liquid water that declined as hydration proceeded; emission also increased with temperature and wounding 12, 13, 14. UPE by seeds is also oxygen dependent 15. When emission wavelengths were determined in hydrated soybean seeds maximum photon emissions were centered at 540 nm and 590 nm over the range 450–630 nm 15. In hydrated rice seeds, photon emission was greater from the seed coat than from the naked caryopsis 13.

The detection of UPE from seeds and seedlings was used diagnostically to look at stress responses during aging and germination 12, 13, 16. Stress responses and aging in seeds are intimately linked with ROS 5, 16, which are also responsible for UPE 1, 2. Although ROS are damaging to plants and linked to seed aging, they have important signaling functions, for example, during dormancy loss by dry after‐ripening (DAR) in dry seeds and during germination 4, 17, 18, 19. However, despite the role ROS play in seeds, the potential of ROS‐derived UPE as indicators of environmental conditions in the soil where seeds persist for long periods has not been investigated. Here we investigate UPE in seeds in response to environmental conditions they are likely to experience in the soil.

When seeds are shed from the mother plant some enter the soil forming the soil seed bank. This persistent seed reserve buffers the population against adverse conditions and *via* dormancy cycling spreads seedling emergence over several years, making dormancy a major component of plant fitness 20. A number of mechanisms are involved in dormancy regulation of which the best understood is the abscisic acid/gibberellic acid balance hypothesis 21, 22. Other mechanisms are also involved such as temperature, nitrate, ethylene and light sensing 19, 23, 24. As dormancy declines seeds appear to become progressively sensitive to environmental signals in the order low temperature, nitrate then light 25.

In the soil seeds are effectively in virtual darkness. Light penetration of soil is rapidly attenuated by covering vegetation and soil depth as a result of soil color, particle size, and moisture content especially at lower wavelengths (<700 nm). Little light of physiological significance is thought to penetrate below 5 mm 26, 27. Therefore, in the soil seed bank any UPE generated by the seed would have low background noise likely overcoming the low signal‐to‐noise ratio dilemma highlighted by Kucera and Cifra 10. Seeds coats further reduce light penetration to the embryo in the phytochrome range by 95% or greater 28. Thus UPE generated within the seed could have a high signal‐to‐noise ratio. Furthermore, plants have evolved highly sophisticated light perception systems (*e.g*. cryptochrome and phytochrome) that perceive light quality and intensity enabling them to detect and respond to the light environment 29. Seeds have co‐opted these systems to integrate sensing of the light environment into the control of dormancy and germination 30, 31.

In seeds, photo‐activated phytochrome does not decay back to the inactive form at low moisture contents 32 which are consistent with those experienced by seeds beneath the soil surface. This suggests a light “memory” can accumulate resulting in a threshold response. These perception systems detect light in the UPE spectral range 33, 34, suggesting seeds may have integrated ultraweak photon emission as a signal that informs of a changing environment.

In this study we investigated UPE from intact seeds, isolated cotyledons and seed coats. We found that seed coat carbonyls likely *via* the formation of triplet excited species were responsible for UPE from the inner layer of the seed coat next to the embryo. This emission was highly responsive to temperature, humidity and oxygen in the range seeds experience beneath the soil surface. This indicates the seed coat has the potential to act as a transducer of temperature, humidity and oxygen signals into UPE. These are key fluctuating environmental variables used as signals by seeds in the soil to time germination and seedling emergence in tune with seasonal and prevailing weather patterns 22.

## Materials and Methods

### Plant material

Dry white Haricot (Cannellini‐Lingot) bean seeds (*Phaseolus vulgaris*, 8% moisture content) were purchased from a local grocery and used as such. Seeds of this type were chosen as the experimental model due to their size and ease of manipulation. UPE was recorded from intact seeds, isolated seed coats or cotyledons. To isolate the seed coat, a dry seed was longitudinally cut in half with a razor blade and the coat gently detached from the underlying embryo (embryonic axis and cotyledons). Only seed coats structurally undamaged as assessed by visual inspection were used. Finally, the poles of the seed coat were excised to avoid curling of the coat after isolation. Seed coats were then weighed and the two half seed coats were put side by side into the sample holder and transferred into the luminometer (Figure S1).

### UPE detection

The luminometer used for the detection of ultraweak photon emission was designed with the capacity to regulate and maintain constant temperature, gaseous fluxes and composition, and relative humidity (RH) in the measuring chamber (see Figure S2 for detailed schemes and description). All the luminometer functions were operated by a series of electronically integrated modules (CREATE PXC‐1011/SCXI, PXI8186 controller, PXI 6070E acquisition board, SCXI‐1112 8‐Channel Thermocouple Input Module, SCB‐68 Shielded I/O connector block, all from National Instruments Co, Austin, TX) controlled by a dedicated LabVIEW (National Instruments, ver 8.2) software module.

Ultraweak photon emission was detected by a photon‐counting device consisting of a low noise 1″ diameter head‐on photomultiplier tube (PMT), high‐voltage power supply circuit and photon‐counting circuit (type num. 7360‐01, Hamamatsu Photonics, Rome, Italy). The PMT had a 300–700 nm detection range with maximal quantum efficiency at 400 nm of approximately 25%. The PMT operated at room temperature with a low dark count rate that increased slightly with increasing chamber temperature within the sample holder (from 18 to 33 counts s^−1^ in the range 20–40°C). Changes in RH and gaseous composition inside the sample chamber had no effect on the dark count.

Long‐pass filters (415, 460, 500, 560, 600 nm; Changchun Worldhawk Optoelectronics Co., Ltd, Changchun, China) were used to measure the spectrum of UPE. The spectral behavior of each filter was verified by recording the light transmission curve in a Beckman DU‐65 spectrophotometer. UPE in the different regions of the spectrum (300–415, 415–460, 460–500, 500–560, 560–600, 600–700) was derived by difference and normalized on the relative PMT quantum efficiency in each region (Figure S3).

Typically, UPE recording was started after 20 min from sample transfer into the reading chamber. Preliminary experiments showed that delayed luminescence resulting from seed manipulation in the light decayed to a minimum in 15 min and did not change for up to 5 h. Basal UPE signals were collected for 5–10 min at the selected reading integration time before starting the treatments. In each experiment, dark counts were subtracted from UPE readings (counts s^−1^). Readings from experiments with isolated coats were then normalized on the sample weight and expressed as counts s^−1^ g^−1^ (samples weights are given in figure legends). Preliminary experiments showed that weight and surface area of isolated coats were linearly related, and a conversion coefficient equal to 55 was calculated. To obtain counts s^−1^ cm^−2^ from counts s^−1^ g^−1^, simply divide the latter by that coefficient. Emission data were analyzed and plotted using Microsoft Excel^®^. Best fits to data were elaborated using Sigmaplot 12 ^®^ (Systat Software Inc). UPE was consistent between seed coats and each trace is representative of at least three experiments. All the experiments were carried out at the Biofluorimetry and Nanoscopy Laboratory of the Department of Experimental Medicine at Sapienza University of Rome, Italy.

### Role of carbonyl groups in ultraweak photon emission

The role of seed coat carbonyl groups in photon emission was investigated by treating isolated coats with 2,4‐dinitrophenylhydrazine (DNPH), a chemical used to qualitatively detect carbonyl functionality in aldehydes and ketones both in solution and on solid supports 35, 36. A positive test was signaled by a pale orange dinitrophenylhydrazone precipitate at the reaction site.

### DNPH treatment of isolated seed coats

A 100 mm DNPH (Sigma‐Aldrich) stock solution was prepared just before use by dissolving DNPH in dimethylsulfoxide (DMSO). A 10 mm DNPH working solution was then prepared by stepwise addition of the DNPH stock solution to water with vigorous stirring.

Isolated bean coats were incubated overnight at room temperature (24°C) in 1 mL of either a 10% (v/v) DMSO solution (control) or 10 mm DNPH working solution. After incubation, both control and DNPH‐treated coats were thoroughly washed in distilled water, wrapped in filter paper and allowed to dry overnight under gentle pressure. On visual inspection of dried samples, only DNPH‐treated coats looked pale orange, indicating the reaction between DNPH and carbonyl groups had occurred.

### Seed coat ultrastructure

For ultrastructural analysis, isolated, unfixed seed coat fragments were imaged with a field emission scanning electron microscope (FESEM, Auriga, Zeiss) at the Research Centre on Nanotechnology Applied to Engineering of Sapienza (CNIS, Sapienza University of Rome).

## Results

### UPE from intact seeds and seed components

**Figure 1:**
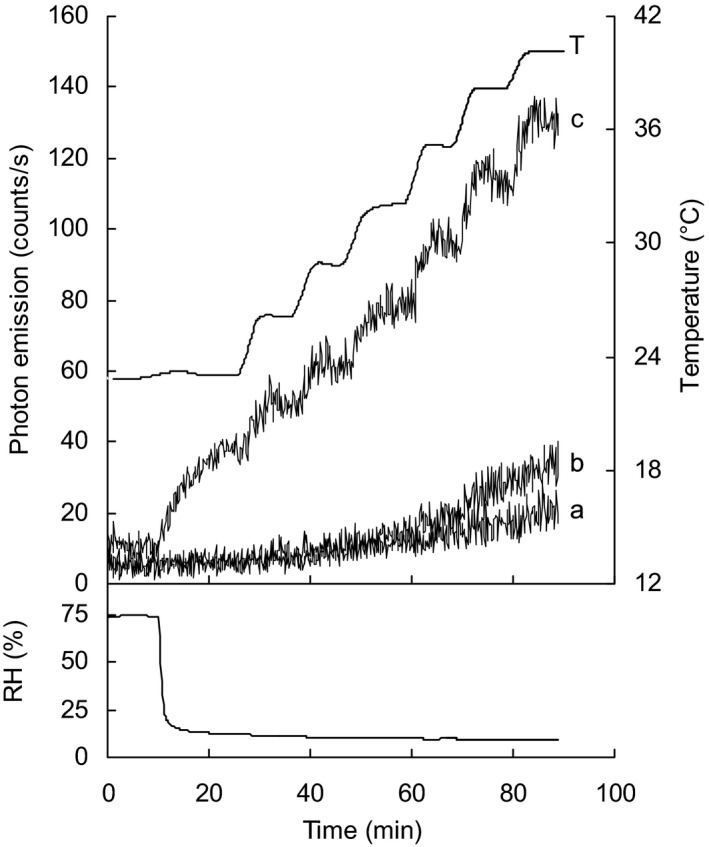
Ultraweak photon emission (UPE) from seed and seed components as temperature increases under low relative humidity (RH) conditions. Following equilibration from 75% to 10% RH, temperature (*T*) was increased in 3°C steps over 90 mins while relative humidity was decreased rapidly after the first 10 mins. UPE is less intense in intact seeds (a) and naked cotyledons (b) than from isolated seed coats (c) with UPE sensitive to both relative humidity and temperature changes. Sample weights were 1100, 450 and 28.5 mg for intact seeds (*n* = 2), naked cotyledons from the same seed (*n* = 2) and isolated half seed coats from the same seed. Data are representative of at least five experiments.

Initial experiments evaluated induced UPE from intact seeds, naked cotyledons and isolated seed coats. At 25°C, UPE from seed coats increased as RH decreased from 75% to 10% (Fig. 1) a range seeds would encounter in the soil. Once equilibrated to 10% RH, stepwise increases in temperature induced a concomitant step increase of UPE from seed coats. Under the same conditions the increase in emission from intact seeds and cotyledons was much less (Fig. 1). Therefore all subsequent experiments tested UPE from isolated seed coats in response to environmental signals.

Mechanical damage to the seed coat incurred during removal from the seed may result in their higher emission. To rule this out UPE was measured before and after damaging intact seeds by introducing three long cuts into the surface of seeds or by repeatedly puncturing them. UPE was measured at 35°C through a cycle from 60% > 20% > 60% RH. No change in UPE was detected between undamaged and damaged seeds (Figure S4).

**Figure 2:**
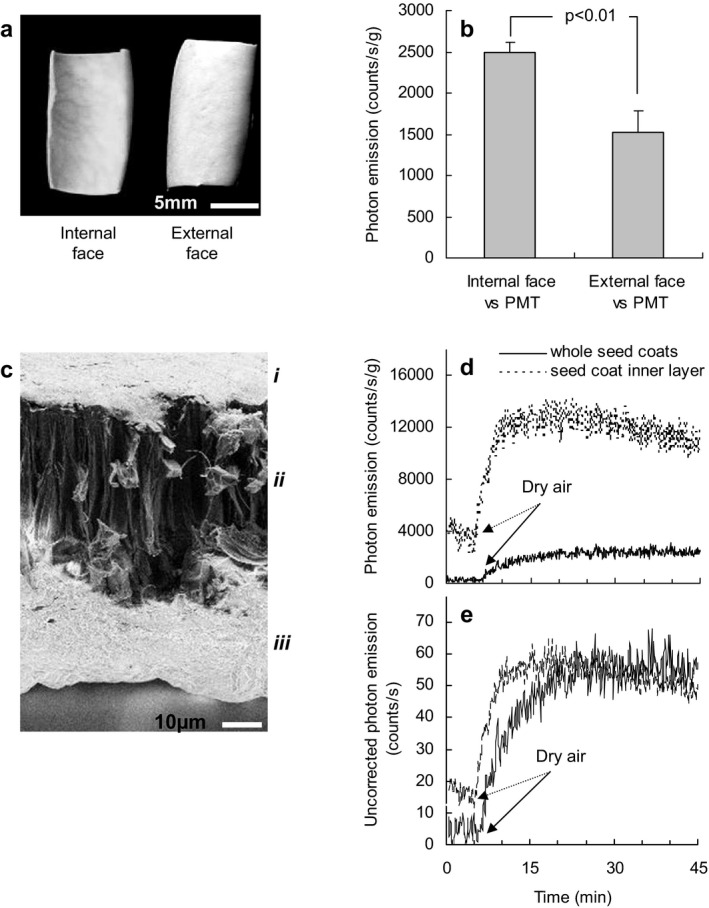
Ultraweak photon emission (UPE) and scanning electron micrograph of seed coats. (a) Top view of seed coats with the internal and external surface shown. (b) UPE from the internal and external side of seed coats at 30°C and 11% relative humidity (RH). Data refer to the photon flux (counts s^−1^ g^−1^) measured after 20–30 min of atmosphere change, that is, when the emission reached a maximum in a stabilized condition (data are mean ± standard deviation (SD); *n* = 3; mean sample weights (±SD) were 33.9 ± 10.8 and 35.2 ± 17.5 mg, for samples placed with the internal or external side toward the photomultiplier tube (PMT), respectively). (c) Field emission scanning electron microscope image of a seed coat transverse section. i, Epidermal layer; secondary thickened (sclerified) palisade cells; ii, subepidermal layer, columnar cells (sclerified); iii, crushed lacunate parenchyma cells of the inner integument (1000×). (d) UPE at 30°C from the internal surface of intact seed coats (continuous line; two half seed coats from the same seed (sample weight 22.6 mg)) and from isolated coat inner layers (dotted line; three pieces from the same seed (sample weight 4.4 mg)). UPE was amplified by dry air admission (11% final RH). (e) Emission data used in (d) uncorrected for sample weight.

The internal or external surface of two half seed coats was separately exposed to the photomultiplier tube (PMT) to determine whether UPE was the same from each face. At 30°C and 11% RH, UPE was statistically higher (*P* < 0.01; Student's *t*‐test, *n* ≥ 3) from the internal surface (Fig. 2a,b). Field emission scanning electron microscopy (FESEM) shows that the inner surface of the seed coat is composed of a thick layer of spongy integument made up of crushed parenchyma cells (Fig. 2c: iii). Under the same conditions of temperature and RH (30°C and 11% RH), we compared UPE of this integument layer detached from seed coats to those still attached to seed coats (*i.e*. as in Fig. 2a). Emission corrected for tissue weight was five times greater from the detached integument (Fig. 2d). When data were not corrected for tissue weight UPE from the detached inner layer is not different from the inner surface of the intact seed coat (Fig. 2e), indicating the former to be the primary source of UPE directed toward the enclosed seed.

The lower UPE from intact seeds compared with isolated seed coats may result from light absorbance by the enclosed seed. Due to the difficulty in obtaining sufficient quantities of the fragile inner integument layer all subsequent experiments used entire seed coats with the internal surface facing the PMT.

### Impact of environmental signals on seed coat UPE

**Figure 3:**
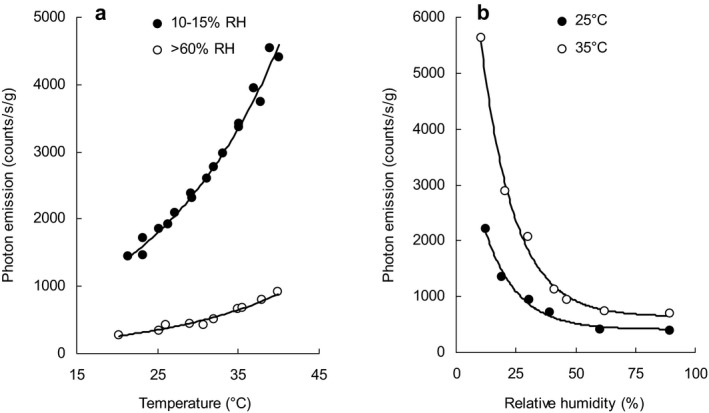
Impact of temperature and relative humidity (RH) on ultraweak photon emission (UPE) from the inner surface of seed coats. (a) UPE from the inner surface of seed coats was recorded at 30 min of intervals at constant temperatures from 20 to 40°C in either low humidity (10–15% RH) or higher humidity conditions (>60% RH). (b) The increase in light emission upon decreasing RH obeys an exponential relationship. The response was similar at 25°C and 35°C (filled and open symbols, respectively), although the effect was much more intense at the higher temperature. Sample weight was between 25.9 and 30.5 mg.

**Figure 4:**
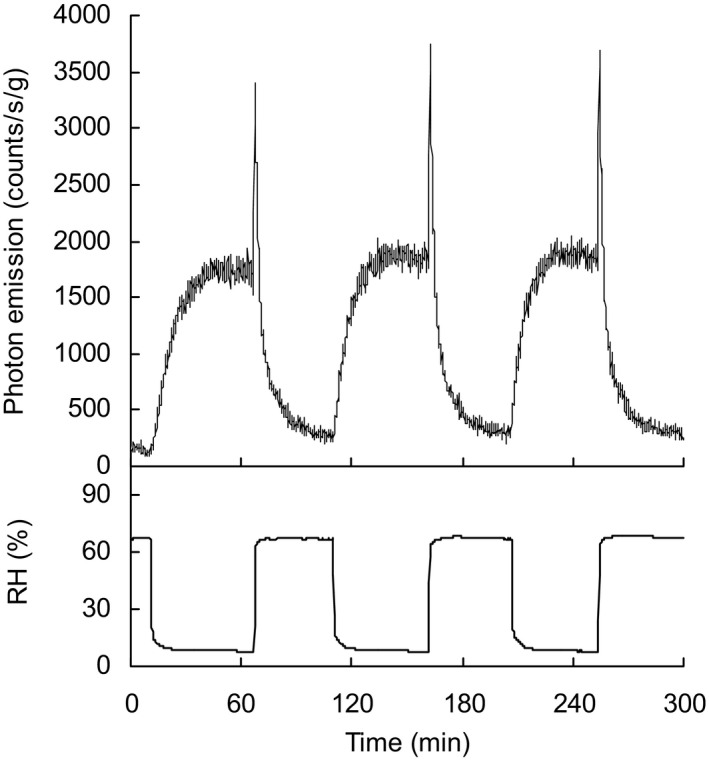
Effect of cyclical changes of relative humidity (RH) on ultraweak photon emission (UPE) from the inner surface of seed coats. Repeated changes from higher (68%) to lower (8%) humidity conditions induce reproducible UPE responses consisting of a progressive increase upon RH decrease, a steep, transient increase upon re‐admittance of humid air followed by an exponential decline to basal levels. Temperature was kept constant at 23°C. Sample weight was 49 mg.

**Figure 5:**
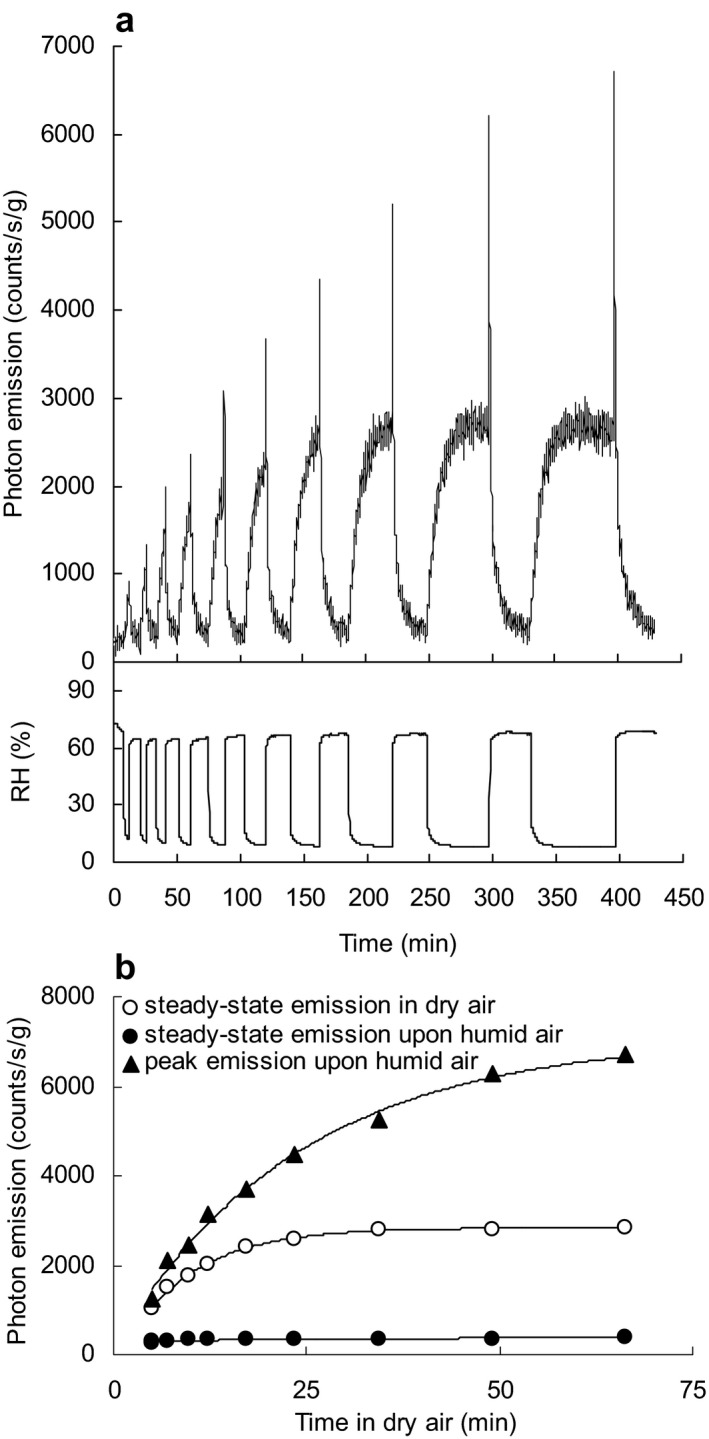
Effect of the duration of dry conditions on ultraweak photon emission (UPE) from the inner surface of seed coats. (a) The longer the dry phase, the higher the UPE peak upon re‐admittance of humid air. (b) Steady‐state emission levels show the humid air‐induced UPE peak continues to increase even after a UPE plateau in dry air was reached. Temperature was kept constant at 30°C. Sample weight was 35 mg.

When emission was measured during stepwise increases in temperature at two different RHs seed coat emission increased with temperature and was greater at low humidity (10–15% RH) compared with higher humidity conditions (>60% RH) (Fig. 3a). Arrhenius plots show the activation energy is similar under both conditions (48 kJ mol^−1^ at low RH and 47 kJ mol^−1^ at higher RH) (Figure S5). This indicates the same mechanism is responsible for the UPE. At constant temperatures (25°C and 35°C) UPE exponentially declined with increasing RH (Fig. 3b). Under dry conditions, re‐increasing humidity from 10% to 65% RH caused a steep emission increase followed by an exponential decrease to basal values (Fig. 4). UPE changes upon RH changes were cyclically reproducible (Fig. 4). When the duration of the dry phase was increased stepwise from 5 to 65 min both the steady‐state emission under dry conditions and peak emission in humid conditions were separately proportional to the duration of the dry phase and indicate saturable components (Fig. 5a,b).

Analysis of the dynamic changes seen in Figs. 4 and 5 reveals that the kinetics of increasing UPE as %RH declined (water vapor desorption) is described by a simple exponential model, with a characteristic time constant of 7.8 ± 0.8 min (*n* = 5), whereas the change in UPE when %RH increases (*i.e*. during water vapor adsorption) is described by a double exponential model with time constants of 1.7 ± 0.4 and 10.9 ± 1.5 min for the fast and slow phases, respectively (*n* = 5). These data indicate that different photon emitters are involved in the two processes.

**Figure 6:**
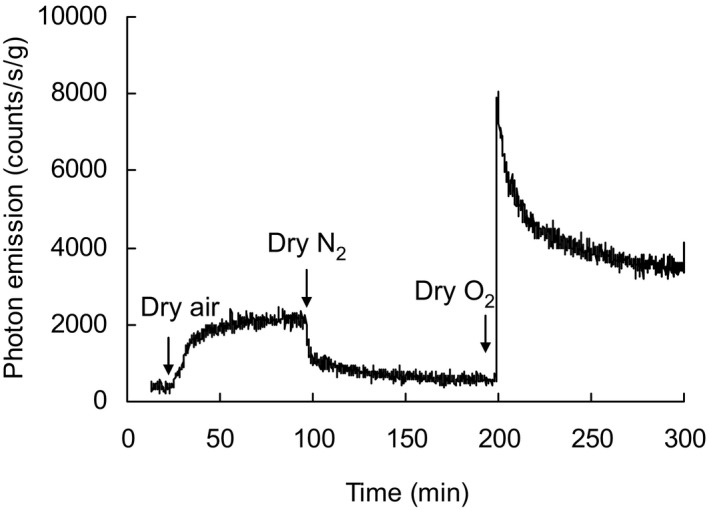
Oxygen dependence of ultraweak photon emission (UPE) from the inner surface of seed coats. UPE was recorded at 27°C and 10–15% relative humidity (RH) as the atmosphere was sequentially changed from air > N_2_ > O_2_. Changes in emission show the strong oxygen dependence of emission. Sample weight was 28.4 mg.

Under dry conditions, when air was replaced with nitrogen, UPE declined two‐fold, and then increased more than eight‐fold when exposed to dry oxygen (Fig. 6). This demonstrates the oxygen dependence of UPE from seed coats.

### Spectral properties of induced UPE from seeds coats

**Figure 7:**
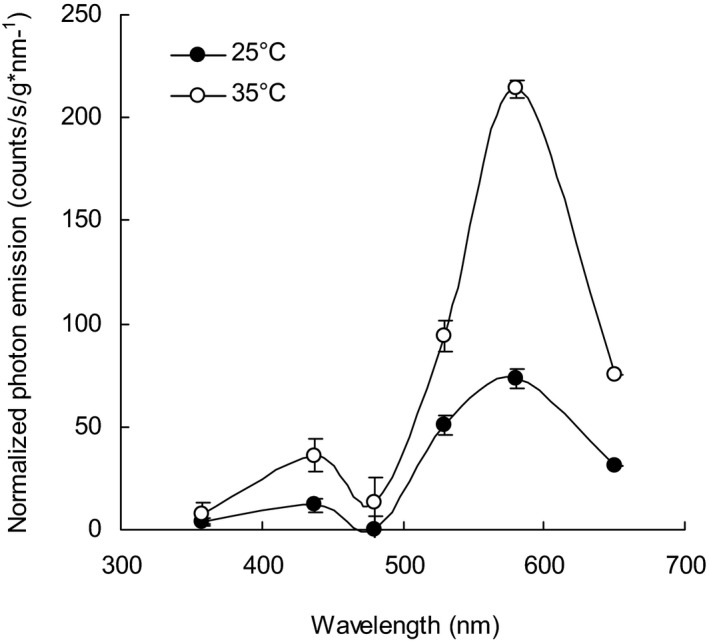
Spectral properties of ultraweak photon emission (UPE). Long‐pass filters were used to analyze the spectra of emission from the inner surface of the seed coat at 9% relative humidity (RH) and either 35°C (open symbols) or 25°C (filled symbols). Two broad peaks of emission are produced, centered in the blue (450 nm) and in the yellow (590 nm) spectral region, respectively. Data are the mean ± standard error (*n* = 6). Sample weights were from 28 to 34 mg. Details for the production of these data are given in the Materials and Methods section and in Figure S5.

Long‐pass filters were used to analyze the photon wavelengths. Under low humidity conditions (10% RH) emissions at 25°C and 35°C were compared. Two temperature‐sensitive emission peaks were observed, one centered on the blue region (450 nm) of the spectrum and another larger one centered on the yellow region (590 nm), encompassing the green to red spectral regions (500–650 nm) (Fig. 7). These peaks were two‐ to three‐fold greater at the higher temperature.

### Carbonyl groups as the source of UPE

**Figure 8:**
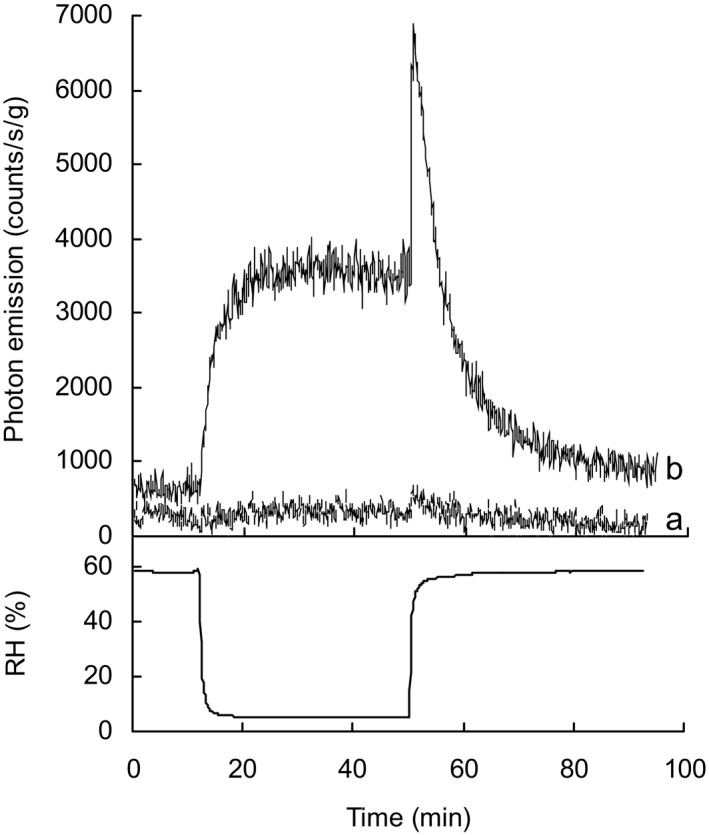
Impact of 2,4‐dinitrophenylhydrazine (DNPH) treatment on ultraweak photon emission (UPE). Seed coats were treated overnight at 24°C with 10 mm DNPH in aqueous solution containing 10% DMSO. Following washing and drying, UPE from treated (a) and control (b) seed coats was measured at 35°C under decreasing and increasing relative humidity (RH). Sample weights were 22 and 23 mg for a and b, respectively.

Ultraweak photon emission from the inner surface of DNPH‐treated and untreated seed coats was measured at 35°C through a cycle from 60% > 10% > 60% RH. Untreated seed coats exhibited the typical increase and spike in emission when RH was cycled. In contrast UPE stayed at background levels in DNPH‐treated seed coats (Fig. 8). This indicates intact carbonyls are needed for UPE from seed coats and suggests a role for triplet excited carbonyls in the phenomenon.

## Discussion

The majority of UPE reports are from hydrated multicellular organisms or cells; here we report emission from the dead tissues of the seed coat, formed during seed development from differentiated cell layers (integuments). Seed coats are highly resistant to environmental degradation in the soil and have a role in the control of dormancy, germination and longevity 37. This persistence of the seed coat suggests a potential role in signal transduction informing the embryo of both rapidly and slowly changing environments.

### Source of seed coat UPE and the response to variable environments

In the present study we show that in the presence of oxygen, the internal layer of *P. vulgaris* seed coats emits photons in response to both increasing temperature and decreasing relative humidity. At maturity the seed coat is composed of dead tissue as confirmed in *Phaseolus lunatus* by viability staining 38. *Phaseolus vulgaris* seed coats are 78% polysaccharide with a lipid component measured as fatty acid of less than 0.2% 39, 40. Both lipids and polysaccharides are sources of UPE with emission from both increasing with temperature 5, 41. Although lipids have emission spectra 41 similar to that seen in this study the very low lipid content of *Phaseolus* seed coats suggests the UPEs in this study are derived from oxidation of seed coat polysaccharides.

Seed coat UPE in response to temperature, oxygen and RH is consistent with that seen in the oxidative degradation of cellulose, with the energy of activation (*E*_*a*_) values for seed coats (47–48 kJ mol^−1^) similar to that for cellulose (55 kJ mol^−1^) at temperatures below 100°C 5, 42, 43. This suggests the cellulose cell walls of the crushed parenchyma that make up the inner integument are the primary source of UPE in this study. Furthermore we show that carbonyls are the originating source of seed coat UPE as blocking carbonyl groups with DNPH effectively eliminated UPE above background levels.

Thus, we hypothesize that the direct interaction of oxygen with electron acceptor moieties in the cellulose structure provides the energy to excite carbonyl groups, which in turn emit light (350–550 nm wavelength range) as they return to the ground state 1, 44. If molecular oxygen or pigments are in close proximity to excited carbonyls (typically less than 10 nm 45), a nonradiative energy transfer can occur with the formation of singlet oxygen (^1^O2) (dimol form) or excited pigments, which then emit at longer wavelengths (550–750 nm) 1, 2. The above interpretation fits well with the results of spectral analysis of seed coat UPE showing two emission peaks, one centered on the blue region and another one centered on the yellow region.

The kinetics of UPE from seed coats under decreasing and increasing relative humidity indicates different mechanisms contribute to photon emission during water vapor adsorption and desorption. The transient, abrupt increase in UPE during seed coat rehydration may result from water‐induced production of singlet oxygen radicals from superoxide anions that accumulate during the dry phase 42. We show that in the presence of DNPH the transient UPE peak seen on rehydration of seed coats is eliminated, indicating that in this case the energy transfer from superoxide to singlet oxygen (or excited pigments) is potentially *via* carbonyls. The rapid phase of UPE decrease would then reflect the decay of this phenomenon whereas the subsequent slower phase (as well as the single phase of UPE increase during water vapor desorption) would instead reflect the effects of water adsorption/desorption onto the coat matrix. Seed coat moisture may modulate UPE intensity in different ways: on the one hand, water vapor adsorption increases polymer chain mobility leading to excited species relaxation *via* nonradiative energy transfer 42, while, on the other hand, water vapor decreases molecular oxygen availability, since at constant temperature and pressure, oxygen partial pressure decreases as %RH increases 42.

All the above suggests that ROS play a crucial role in seed coat UPE. Cellulose is degraded by the oxidative action of ROS 5, 42, 43 under conditions similar (temperature, RH, oxygen) to those seeds encounter in the soil, suggesting that seed coat weakening may be environmentally driven. This complements the role of ROS in loss of dormancy during dry after‐ripening, signaling during early imbibition and endosperm loosening during germination 4, 18, 46. The dependence of UPE from the seed coat on oxygen indicates this only occurs near the soil surface in noncompacted soils as oxygen levels decline sharply with distance below the soil surface.

### Seed and UPE response to seedbed environmental signals

The conditions used in this study are within the physiological range of the environmental signals seeds experience in seasonally variable soil environments 22, 47. In particular, the range of changes in temperature and water availability (RH) represents rapid local changes experienced beneath the soil surface when seeds undergo hydration/dehydration cycles. Water availability in the soil surface layers varies greatly and the seed, in particular the seed coat, rapidly equilibrates to its surrounding conditions. In the present work, the moisture content of the seed coat rapidly comes into equilibration with ambient RH and is representative of seed coat drying in the surface layers of the soil. This is important as in unsaturated soils, water vapor is the predominant source of water taken up by seeds 48.

The seed coat is the first seed layer to experience changes in the soil environment. The outer integuments of the seed coat are water and oxygen impermeable to varying degrees with exchange of air and water occurring *via* the hilum and micropyle. These structures are in close proximity to the embryonic axis/radicle tip where growth leading to germination completion is initiated. In *P. vulgaris*, changes in moisture content first occur in the hilum region and associated testa before the underlying radicle and cotyledons 49.

Ultraweak photon emissions from intact seeds are low and relatively unvarying in response to variable temperature and RH levels in comparison with the detached seed coat. It could be argued that coats in intact seeds, as compared to isolated coats, equilibrate at a lower rate which contributes to this difference. Furthermore, in intact seeds UPE from the inner layers of the seed coat may be absorbed either by the lignified outer seed coat layers or by the enclosed seed also contributing to the above difference. Notably, the seed contains many air spaces typically in the crushed parenchyma layer of the seed coat and between the seed coat and the underlying seed in the vicinity of the hilum 50, 51. As air and water exchanges occur through the hilum these air spaces favor a rapid equilibration with the external environment. This would allow UPE from inner integuments of the coats in the hilum region to respond quickly to environmental changes. As a result changes in temperature‐ and humidity‐related UPE could then be detected by the embryonic axis/radicle tip that initiates growth in this nonendospermic species.

In endospermic species the endosperm (aleurone) layers that surround the embryo in particular the micropylar endosperm cap that covers the radicle tip, exist immediately below the seed coat. The behavior of this cap is consistent with it being the principal receptor for environmental signals that control of germination 46, 52, 53. Thus environmentally driven changes in UPE on the underside of the seed coat are ideally placed to influence this control.

### Potential role of seed coat ROS and UPE in environmental sensing

As seed dormancy cycles in the dark of the soil seed bank, slow seasonal change (temporal sensing) driven by temperature alters the depth of dormancy. When dormancy is lowest, seed germination potential increases with the seed becoming increasingly sensitive to environmental signals (spatial sensing) that inform the seed of its depth in the soil (diurnal temperature difference, oxygen) and the presence of competing plants (light, temperature, nitrate) 22, 54, 55. As dormancy declines to low levels (spatial sensing phase), seeds of some species go from being light insensitive to highly sensitive 22, 47, 56, 57. It is during the spatial sensing phase that the ROS‐driven process of dormancy reduction by dry after‐ripening may have a significant role due to low soil moisture 22. We suggest it is at this point in the annual dormancy cycle that seeds have the potential to perceive ROS‐generated UPE. This would provide another complementary route for the involvement of ROS, beyond direct ROS signaling 4, 17, 18, 19, to regulate dormancy and the completion of germination.

### Do seeds have a mechanism capable of detecting UPE?

Seeds contain both cryptochrome and phytochrome that can detect the blue and yellow parts of the spectrum we show for peaks of UPE from the seed coat. Of the phytochromes, we propose PHYA is a candidate sensor for UPE. In seeds, PHYA is the most abundant phytochrome, accumulates to high levels in the dark 58 and photo‐irreversibly induces germination in monochromatic light from 300 to 770 nm 33 covering the UPE spectral range. Furthermore at low seed moisture contents (0.086–0.11 g H_2_O g^−1^ dry weight), active phytochrome formed by red light (660 nm) survives further desiccation and on hydration induces germination 32. In the context of PHYA other wavelengths may produce a similar response. Below a moisture content of 0.1 g H_2_O g^−1^ dry weight dormancy loss is associated with nonenzymatic generation of ROS 59 and we show high UPE at these moisture contents where active phytochrome can be formed (see above). This provides a mechanism whereby the pool of active PHYA can increase toward threshold levels due to the impact of ROS‐derived UPE during hydration/dehydration cycles beneath the soil surface. A key question is whether UPE provides sufficient light to be effective. In support of this it has been shown that oat seedling phytochrome can be photo‐converted to the active form by UPE from excited carbonyls 60.

Beneath the soil seeds are effectively in perpetual darkness at a depth of ~5 mm depending on soil type and vegetation cover 26, 27, indicating UPE may be the predominant light source. Another below‐ground variable is the requirement of oxygen for UPE. In noncompacted soils gaseous diffusion through soil is constant with depth (*e.g*. to a depth of 45 cm in clay soil), and is highest in dry soils (low water potential) decreasing rapidly as the pore structure becomes water filled or reduced as field capacity is approached (high water potential) or as soil is compacted. The oxygen content of soil air is also relatively constant at 19–20% to a depth of 15 cm 61, so oxygen availability would not be limiting in the absence of soil compaction.

When light sensitivity increases in the spatial sensing phase dormancy can be broken by millisecond flashes of low‐fluence sun light. This is PHYA mediated and saturated by <1% of active phytochrome 57, 62 with up to 80% of buried *Datura ferox* seeds responding to <0.01% active phytochrome 28. Dark incubation of seeds sensitized them to dormancy breaking by PHYA‐mediated low‐fluence red light in the range 1–100 nmol m^−2^ s^−1^. In the same study a 50% germination threshold was crossed by fluence rates from 0.1 to 10 μmol m^−2^ s^−1^ at wavelengths from 300 to 560 nm 33. With seed coat attenuation of transmitted light in the phytochrome range of 95% or greater 28 the effective fluence rate without an intervening seed coat would be even lower.

In the case of UPE the proximity between the emitter (inner seed coat) and receiver (radicle tip/micropylar endosperm cap) may provide the required intimacy to overcome fluence rate response thresholds. In etiolated seedlings a response threshold of 10 pmoles m^−2^ s^−1^ was measured 63, which is five orders of magnitude higher than the UPE measured in this study. These measured thresholds may also be artificially high due to technical limitations in the measurement of light levels and the actual thresholds of sensors within the cell. For example in Adamska 63, fluences lower than the hypothetical threshold value (10 pmol m^−2^ s^−1^) were not tested and the instrument used was unable to measure fluence values less that few dozen pmol m^−2^ s^−1^.

Finally the potential involvement of heterotrimeric G proteins in PHYA‐mediated signaling and germination 64, 65 provides a mechanism for signal amplification similar to that in retinal rod photoreceptors where heterotrimeric G proteins enable signal amplification from single photons into a response 66. Taken together these observations form a convincing argument that PHYA may be able to perceive and respond to UPE so contributing to the environmental regulation of seed germination.

## Conclusions

Seeds have evolved to be highly specialized environmental sensors in order to adjust dormancy status and germination potential to their immediate and long‐term environments. In the results presented here we demonstrate that the biophysical properties of seed coats have the potential to function as transducers of temperature, relative humidity and oxygen signals over the range seeds experience in the soil seed bank. This presents a sensory mechanism potentially operating in parallel with other sensing mechanisms controlling the seeds’ response to the environment. If environmentally sensitive regions such as the micropylar endosperm and radicle tip can detect UPE from the seed coat during cycles of wetting and drying within the soil seed bank it means they are far more intimately in tune with the environment than previously envisaged.

## Supporting information


**Figure S1.** Picture of the sample holder unit with two half‐seed coats put side by side for UPE recording.
**Figure S2.** Schematic representation of luminometer and related equipment.
**Figure S3.** PMT sensitivity curve as modified by the long‐pass filters used for UPE spectral analysis.
**Figure S4.** Effect of seed coat damage on photon emission from intact seeds.
**Figure S5.** Arrhenius plots showing the activation energy (*E*_*a*_) of photon emission from isolated seed coats.
