# Supplementary material for: Ultraweak Photon Emission from the Seed Coat in Response to Temperature and Humidity—A Potential Mechanism for Environmental Signal Transduction in the Soil Seed Bank
Source: Photochem Photobiol. 2016 Aug 19;92(5):678–87. doi: 10.1111/php.12616 (PMC5031227; doi:10.1111/php.12616)
Supplement: Supplementary file 1 — Figure S1. Picture of the sample holder unit with two half‐seed coats put side by side for UPE recording. Figure S2. Schematic representation of luminometer and related equipment. Figure S3. PMT sensitivity curve as modified by the long‐pass filters used for UPE spectral analysis. Figure S4. Effect of seed coat damage on photon emission from intact seeds. Figure S5. Arrhenius plots showing the activation energy (E a) of photon emission from isolated seed coats. [file PHP-92-678-s001.pdf]

SUPPORTING INFORMATION

Figure S1

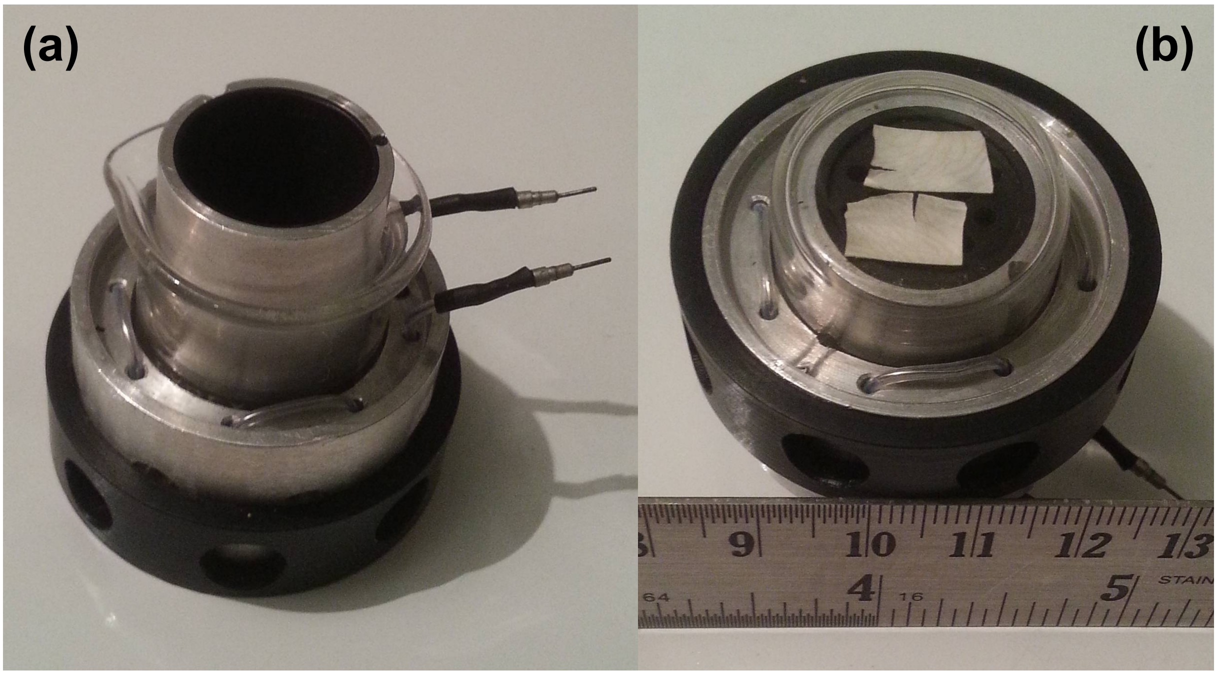

Figure S1. Picture of the sample holder unit with two half-seed coats put side by side for UPE recording.
(a) The sample holder unit (top view)
(b) Two isolated coats inside the holder (bottom view).

Figure S2

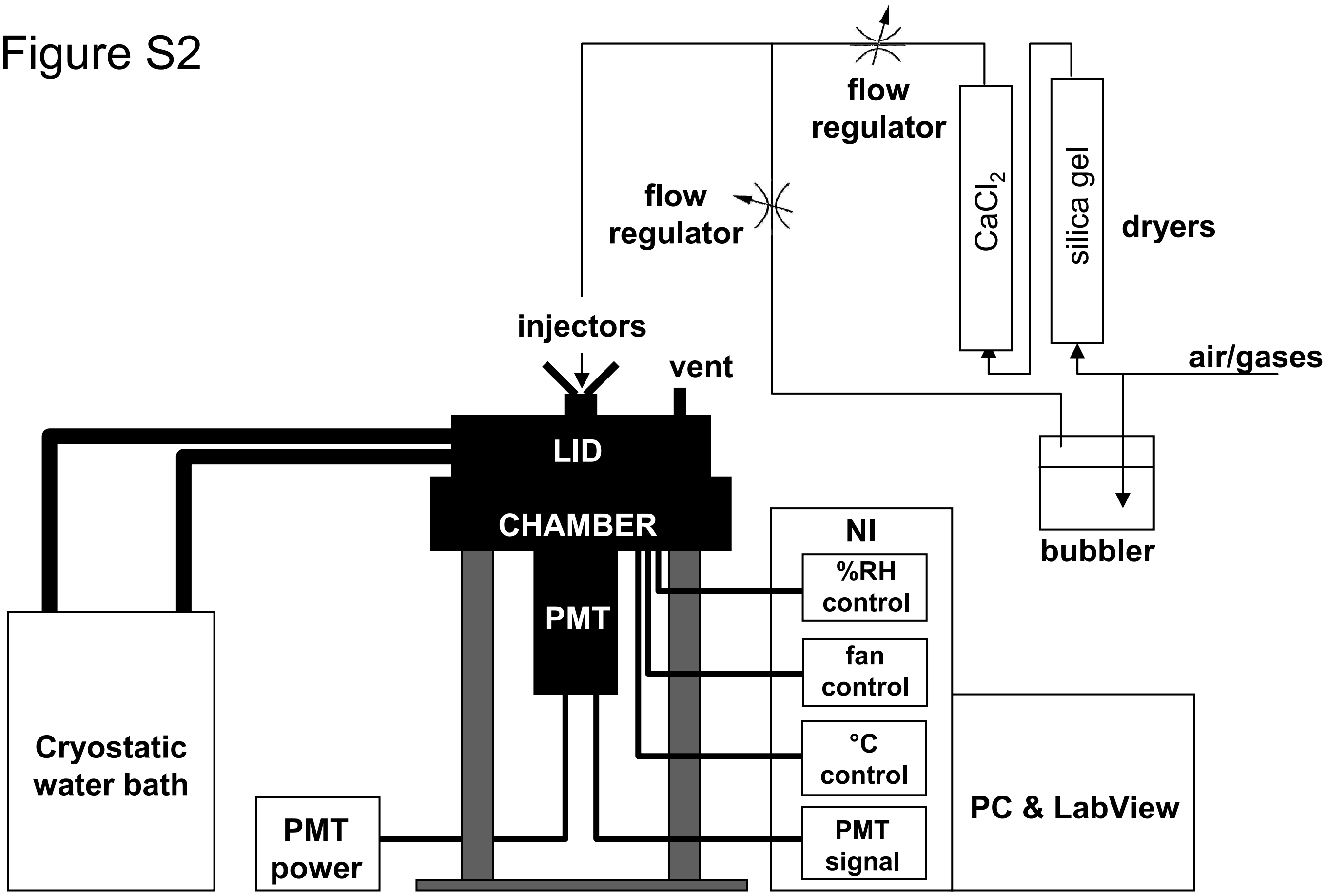

flow regulator
flow regulator
CaCl$_2$
silica gel
dryers
injectors
vent
air/gases
LID
CHAMBER
bubbler
NI
%RH control
PMT
fan control
°C control
Cryostatic water bath
PC & LabView
PMT power
PMT signal

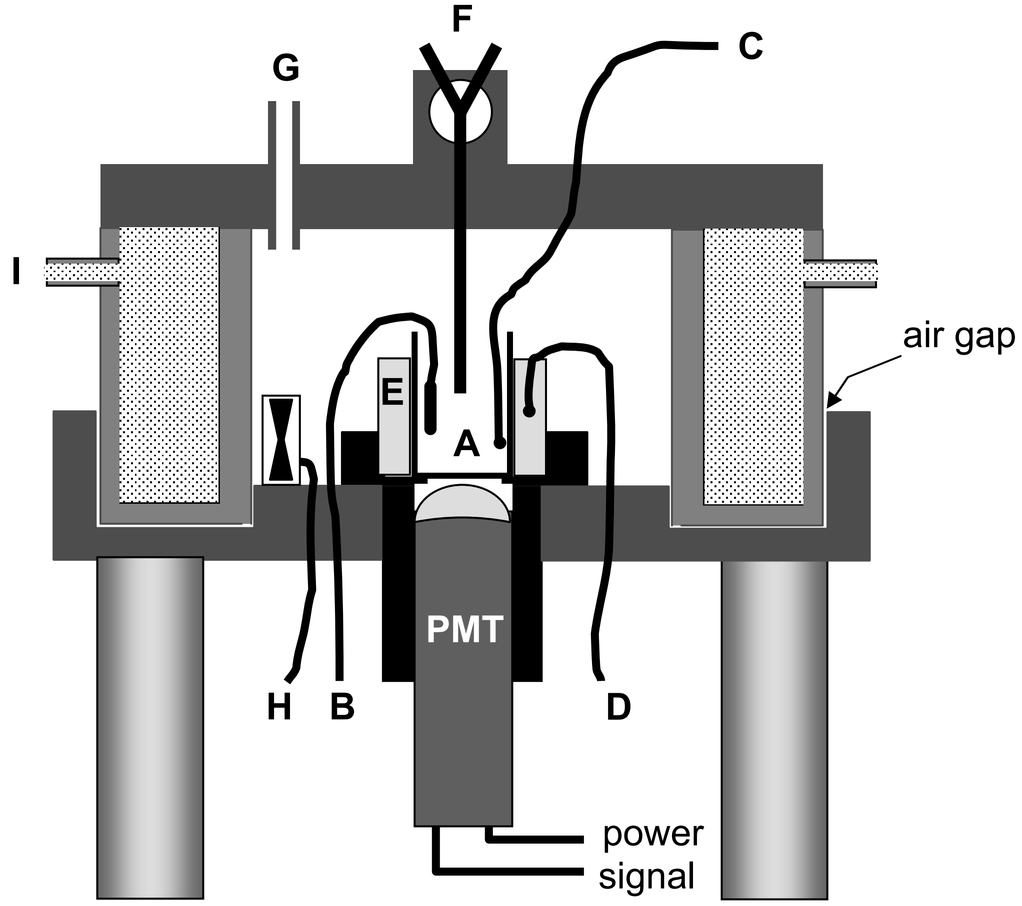

F
C
G
I
air gap
E
A
PMT
H
B
D
power
signal

Figure S2. Schematic representation of luminometer and related equipment. System overview (upper panel) and cross section of the chamber (lower panel). The luminometer consists of a two-part circular structure machined from black PVC. The lower part has a central plinth with a hole to engage the photomultiplier tube (PMT) holder. The PMT holder consists of a hollow PVC cylinder jutting 2 cm over the plinth and protruding 5 cm below the chamber bottom. The PMT is slid in from below and secured in place. That part of the PMT holder extending into the chamber is indented to hold the specimen 4 mm away from the PMT photocathode (for PMT characteristics see text).

The sample holder (A) consisted of a 12 ml capacity borosilicate glass (Pyrex) beaker. Light transmission properties of the sample holder were checked with a Beckman DU-65 spectrophotometer and found to be >80% in the PMT sensitivity range.

The upper part (lid) of the luminometer, has an inner and outer wall bearing two hose adapters for water circulation (I). An air vent in the lid (G) enables the composition of the atmosphere to be modulated inside the luminometer. To prevent the entry of light after passing through the lid, the vent tube is coiled to produce a 2-turn spiral with the open end turned upwards, almost in contact with the internal lid surface. This air vent allows slow, gradual atmosphere changes in the luminometer chamber. In this study (G) was kept plugged and atmosphere changes were via a two-way injector system in the lid (F) allowing liquid/gas to be delivered into the sample chamber, near to the specimen, by hand-operated syringes (liquids) or controlled gaseous fluxes.

The atmosphere composition inside the luminometer was controlled by pump forced fluxes (1-3 L m-1) of ambient air, nitrogen or oxygen. Humid or dry atmosphere (about 90% and 10%RH, respectively) was obtained by either extensively bubbling air/gases in distilled water or by passing them through coupled granular CaCl2 and silica gel columns, respectively. Ambient air at different %RH was obtained by mixing different percentages of humid and dry air. Nitrogen and oxygen were obtained from standard N2 and O2 cylinders, respectively.

Humidity (%RH) in the proximity of the sample was monitored with a humidity probe (B) inside the sample holder (A). A thermocouple (C) inside the sample holder (A) probed the specimen’s temperature and another thermocouple (D) in the sample holder heater (E) drove a thermostating electric circuit to maintain a constant temperature (+/- 0.3 °C). The temperature inside the detection chamber was controlled (+/- 0.5 °C) by a thermostated external circulating water bath connected to the lid (I). A small fan (H) continuously circulated the internal atmosphere. Operation of the fan did not interfere with PMT output.

Once assembled, the geometry of the upper and lower sections ensured the exclusion of external light while a small air gap (less than 0.5 mm) between them allowed the internal and external pressure to equilibrate.

Figure S3 Spectral analysis

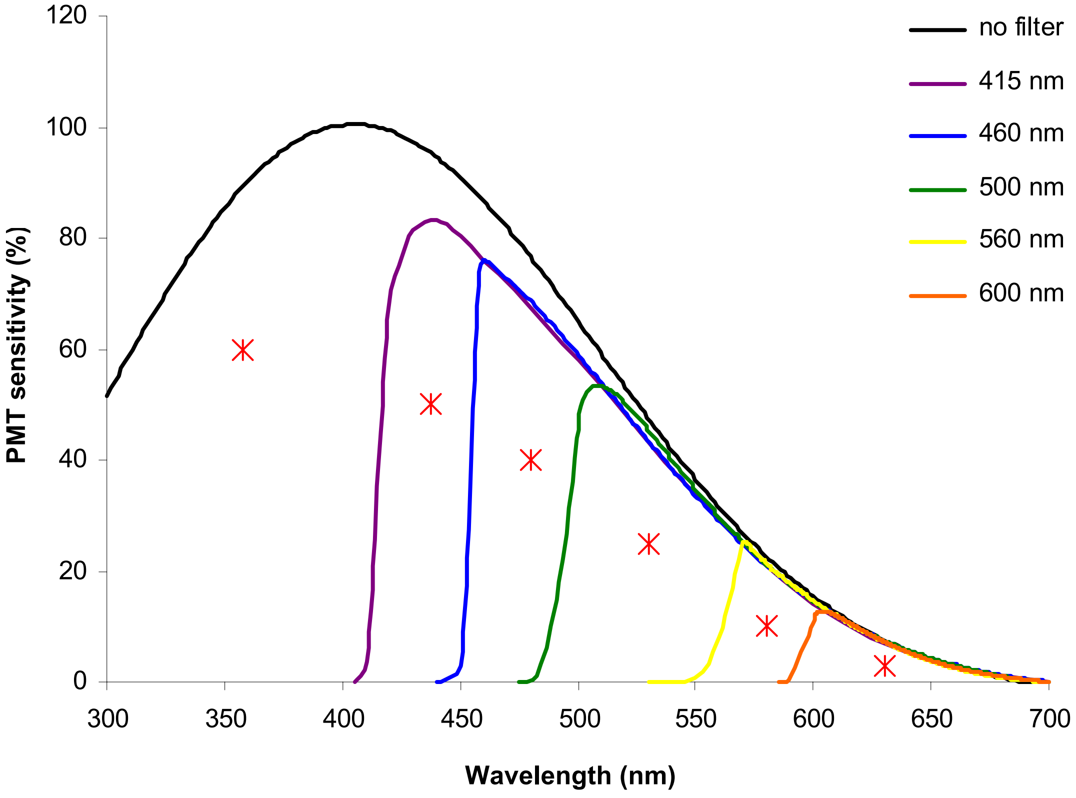

Figure S3. Based on the long-pass filter transmittance properties, modifications of PMT spectral sensitivity curve were established and the area under the curve (AUC) in the absence and presence of each filter was calculated (Total Detectable Optical Power, TDOP).

Sample photon emission was separately obtained in absence and presence of each filter.

Different regions of the light spectrum were identified (red asterisks) and photon emission and TDOP in each region were derived by difference.

Normalized photon emission was then calculated by dividing the differential photon emission by the corresponding differential TDOP.

Example:
The normalized photon emission in the 415-460 region was calculated as follows:

(sample counts measured with the 415 nm filter – sample counts measured with the 460 nm filter) / (TDOP as modified by the 415 nm filter - TDOP as modified by the 460 nm filter)

Figure S4

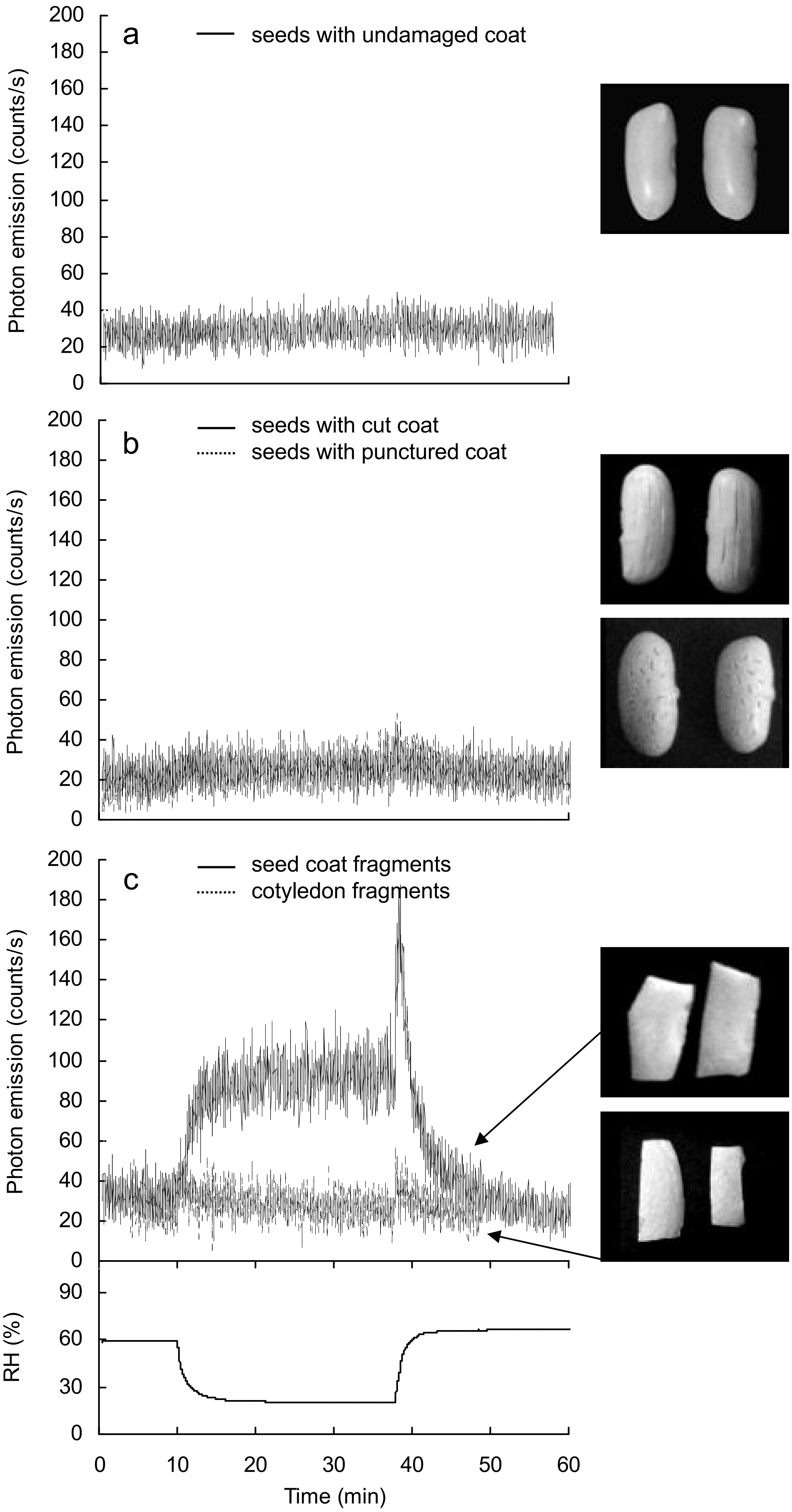

Figure S4. Effect of seed coat damage on photon emission from intact seeds. Photon emission was measured at 35°C through a 60-20-60 %RH cycle in intact seeds (a), seeds with damaged coat (b), isolated coats and naked cotyledons (c). Sample weights were 1000-1200, 390 and 32.7 mg for whole seeds (2X for each trace), naked cotyledons (2 fragments from 1 seed) and isolated seed coats (2 fragments from 1 seed).

Figure S5 Arrhenius plots

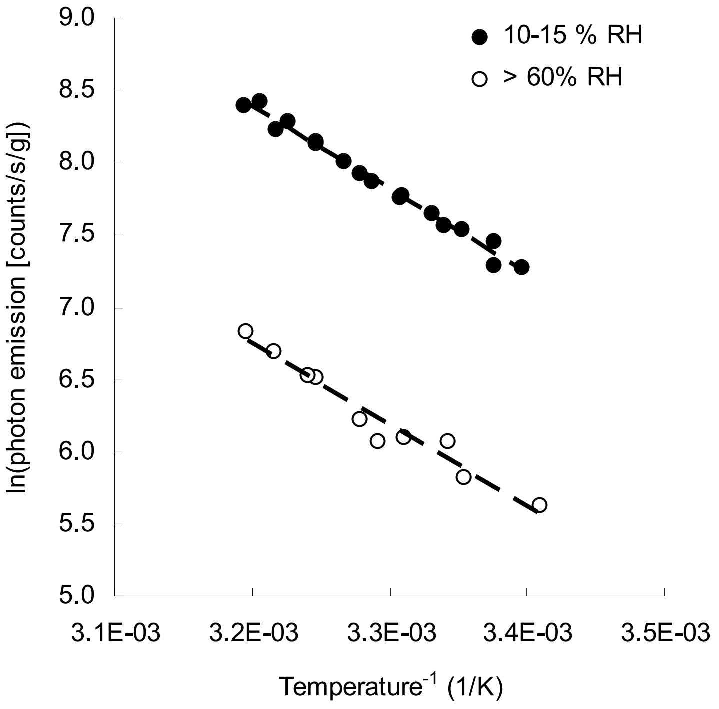

Considering the Arrhenius equation:

$\ln(k) = -E_a/R\ (1/T) + \ln(A)$

where k = the rate constant of a chemical reaction
T = temperature (K)
E_a = activation energy
R = universal gas constant, value $8.314 * 10^{-3}$ kJ mol^{-1} K^{-1}
A = constant factor depending on the nature of the chemical reaction

and assuming
k = photon emission intensity (luminescence intensity is proportional to the rate constant of the chemical reaction producing excited, emitting molecules)

Arrhenius plots were constructed by using coat UPE measured at two%RH (10-15% e >60%)

For each plot, the best fit was calculated according to the equation Y = a + b*X and the slope value (b) was used to derive the activation energy (E_a) according to the formula:

E_a (kJ*mol^{-1}) = -b*R

Figure S5. Arrhenius plots showing the activation energy (Ea) of photon emission from isolated seed coats. The activation energy of emission (represented by the slopes of the curves) is similar in dry (filled symbols) and humid air (open symbols), indicating that the same mechanism is responsible for the emission. The procedure for Ea calculation is also showed.
